# *Bacillus subtilis* CBR05 induces Vitamin B6 biosynthesis in tomato through the *de novo* pathway in contributing disease resistance against *Xanthomonas campestris* pv. *vesicatoria*

**DOI:** 10.1038/s41598-019-41888-6

**Published:** 2019-04-24

**Authors:** Murugesan Chandrasekaran, Manivannan Paramasivan, Se-Chul Chun

**Affiliations:** 1 Department of Food Science and Biotechnology, Sejong University, 209 Neungdong-ro, Gwangjin-gu, Seoul 05006 Republic of Korea; 2 Department of Microbiology, Bharathidasan University, Tiruchirappalli, 620024 Tamilnadu India; 3 Department of Environmental Health Science, Konkuk University, 120 Neungdong-ro, Gwangjin-gu, Seoul 05029 Republic of Korea

**Keywords:** Environmental microbiology, Biotic

## Abstract

Expression profiling for genes involved in Vitamin B6 (VitB6) biosynthesis was undertaken to delineate the involvement of *de novo* and salvage pathway induced by *Bacillus subtilis* CBR05 against, *Xanthomonas campestris* pv. *vesicatoria* in tomato. Pyridoxine biosynthesis (PDX) genes such as *PDX1.2* and *PDX1.3*, were found to be overexpressed significantly at 72 hpi in *B. subtilis* and pyridoxine inoculated plants. Most significant upregulation was observed in the transcript profile of *PDX1.3*, which showed more than 12- fold increase in expression. Unfortunately, salt sensitive overlay4 (*SOS4*) profiling showed irregular expression which corroborates that *SOS4* role in VitB6 biosynthesis needs further studies for deciphering a clear notion about their role in tomato. Antioxidant enzymes i.e., superoxide dismutase, catalase, polyphenol oxidase, and peroxidase activities clearly demonstrate escalation till 48 hpi and gets reduced in 72 hpi. Pot trials also confirm that *B. subtilis* compared to pyridoxine supplementation alone show plant disease resistance and elongated roots. The present study confirms that *B. subtilis*, as a versatile agent in eliciting induced systemic resistance regulated by *de novo* pathway as a model for plant defense against *X. campestris* pv. *vesicatoria* substantiated by VitB6 biosynthesis. Nevertheless, the study is preliminary and needs further evidence for affirming this phenomenon.

## Introduction

The cofactor Vitamin (VitB6) is a combination of six water-soluble vital vitamers with a common pyridine ring composed of variations in 4′ moieties possessing alcohol pyridoxine (PN), amine pyridoxamine (PM), aldehyde pyridoxal (PL) and 5′ phosphorylated forms (PNP, PMP, PLP)^1^. The PLP vitamer plays an essential role as an enzymatic co-factor in more than 140 biochemical reactions and has also recently been implicated in defense against cellular oxidative stress. In plants, two different pathways such as *de novo* and *salvage* pathway have been described for biosynthesis of vitB6 vitamers^2–6^. First, the deoxy-xylulose 5-phosphate (DXP) independent pathway was used for *de novo* biosynthesis of vitB6 vitamers^4^. It involves the concerted activities of the pyridoxine biosynthesis proteins (PDX) such as pyridoxine biosynthesis protein1 (PDX1) and 2 (PDX2), that form a multimeric protein complex to synthesize pyridoxal-5′-phosphate (PLP) as an active cofactor^2–5^. Second, the salvage pathway predominantly is responsible for the conversion of vitamer forms through specific enzyme modificiation^6,7^. It converts, PN, PM, and PL to active co-factor PLP by the concerted activities of a vitB6 kinase (salt overlay sensitive 4 (SOS4)) through phosphorylation while biosynthesis of PLP from PN and PM requires the activity of a vitB6 oxidase (PDX3)^8,9^. Recently, a pyridoxal reductase has been characterized in *Arabidopsis thaliana*, which is essential for conversion of PL into PN^10^.

Studies showed that abiotic stress response comprises of pyridoxal kinase and pyridoxal reductase enzymes which play a significant role mediated by salvage pathway^8–11^. Previous studies also showed that abiotic stress regulates up-regulation of *de novo* biosynthetic pathway genes^12–17^. *A*. *thaliana* (*AtPDX1*) gene mutation renders the plants for the high rate of sensitivity to stress conditions like high light, salt and stress. Nevertheless, *PDX* gene overexpression provides escalated tolerance in combating oxidative stress^16,17^. The gene for pyridoxal kinase, *SOS4* which is a key factor in the salvage pathway was alienated to sequential response to salinity and osmotic stress^8–11^.

Moreover, previous studies clearly depict salicylic acid (SA), methyl jasmonate (JA), and ethylene (ET) act as the chemical counterparts in inducing plant defense response abating oxidative stress through overexpression of *PDX* transcript in *Nicotiana tabacum* and *Hevea brasiliensis*^18,19^. The studies lucidly show that the hypersensitive response (HR) in tobacco leaves infected with *Pseudomonas syringae* pv. *phaseolicola* was affected with excess VitB6 content, rather *P*. *syringae* pv. *tabaci* infection resulted in augmented disease severity^18^. However, direct molecular evidence supporting a role for VitB6 in biocontrol agent, *Bacillus subtilis* inoculated plants and their plant biotic defense remains lacking. The present study was focused to analyze the expression profiling of *de novo* (*PDX1*.*2*, *PDX1*.*3*, *PDX2*), and salvage pathway (*SOS4*) genes to demarcate involvement of *de novo* and salvage pathway in VitB6 biosynthesis by *B*. *subtilis* CBR05 upon challenge emancipated by *Xanthomonas campestris* pv. *vesicatoria* (XCV) in tomato.

Among plant diseases encountered worldwide, bacterial spot disease, caused by XCV pose a serious threat owing to complex pathogen variability which affects economically important crops production^20–25^. Bacterial diversity poses additional threats to biocontrol efficacy of various agents of antagonistic control and inadequacy of effective strategies to gain disease resistance and efficient abatement through chemical control^26^. Besides, restrictions on the use of chemical pesticides due to concerns about their impact on the environment and human health are increasing rapidly^27^. Biological control of disease using microbial antagonists is an eco-friendly alternative to chemical pesticide and is being studied extensively on several different plant diseases^28–32^. Strains of the Gram-positive bacterium, *B*. *subtilis* have been regarded to protect plants opposing fungal and bacterial pathogens. *B*. *subtilis* has been regarded as a versatile bacterium possessing plant growth promotion along with enhanced crop protection mediated by ISR (induced systemic resistance)^30,31,33^. Elicitation of ISR by these strains has demonstrated importance in greenhouse or field trials^34–38^. Mode of action by which *B*. *subtilis* instilling broad-spectrum antagonistic activity against various phytopathogens is attributed to the ability to produce spores, antibiotic production, lytic enzymes and capacity to resists adverse environments^39–41^. Thus, it is clearly evident that *Bacillus* sp. is an essential regulator of ISR.

*B*. *subtilis* is also a predominant endophytic bacterium which marks it as an efficient biocontrol agent against vascular pathogens^28,42^. Endophyte colonization triggers the reprogramming of the host action, favoring secondary metabolism and inducing changes in the plant development^43^. For example, *B*. *subtilis* strain confers protection to melon plants encountering the cucurbit powdery mildew by activating SA and JA dependent defense response^44^. Moreover, they produce the volatile compound such as acetoin, which triggered the ISR. In *A. thaliana*, *B*. *subtilis* UMAF6639 restricts pathogen dissemination and disease progression in its aerial parts through ET- and SA-dependent and JA-independent response^45^. Cyclic lipopeptides such as iturins and fengycins from *B*. *subtilis* acting as potent antibiotics in the biocontrol of the tomato wilt disease caused by the phytopathogenic bacterium *Ralstonia solanacearum*^46^. A change in a JA receptor resulted in the sufficient signal transduction, fertility and defense conflicting insects, in addition to resistance in opposing *P*. *syringae* pathogenic strains^47^. Hence, the recent identification of host target modification could be a promising approach to protect plants from pathogen attack. Thus *B*. *subtilis* acts a significant antagonist in field conditions both in the external environment and endophytic colonization thereby contributing to ISR in eliciting a host defense response for increased productivity and devoid of loss due to phytopathogens,

VitB6 possess antioxidant activity and can modulate plant defense by regulating antioxidant status in plants^48^. However, collaborating VitB6 biosynthesis with biocontrol, disease resistance, ISR, stress alleviation, and tolerance is still absent with regard to *B*. *subtilis* CBR05 against XCV. In the present study, we examined *B*. *subtilis* CBR05 induced VitB6 biosynthetic genes in tomato confronted with XCV. Earlier up to the literature collected it was positively corroborated this is the first report involving *B*. *subtilis* CBR05 induced expression of VitB6 biosynthetic genes in tomato against XCV apart from biocontrol proficiency, ISR and plant improvement based on growth characters. We affirm that the present study could be rationalized for similar diseases not only in tomato but can be extended to other crops belonging to the *Solanaceae* family. The study would be a launch pad in further characterization and involvement of crosstalk between the gene regulation of *de novo* and salvage pathway in ISR mediated by *B*. *subtilis*.

## Results

### VitB6 biosynthetic genes expression and changes in VitB6 content

**Figure 1:**
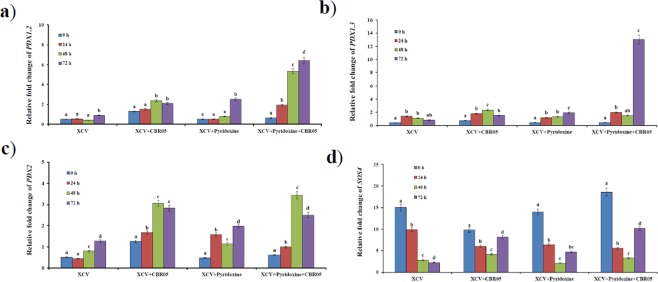
Relative fold-change expression of vitamin B6 biosynthetic genes in *Xanthomonas campestris* pv. *vesicatoria*-infected tomato plants. Quantitative real-time PCR analysis revealed *de novo* genes, including (**a**) *PDX1*.*2*, (**b**) *PDX1*.*3* and (**c**) *PDX2* and salvage pathway gene, (**d**) *SOS4*. Values were normalized to the values of the mock control. Data are means of triplicates ± SD and different letters are significant differences at *P* ≤ 0.05 level.

Expression profiling patterns of both *de novo* (*PDX1* [*PDX1*.*2* and *1*.*3*] and *PDX2*), and salvage (*SOS4*) pathway genes in tomato plants after infection with XCV was undertaken as the primary rationale of the present study. *PDX1*.*2*, *PDX1*.*3*, *PDX2*.*0*, and *SOS4* gene expression levels were compared at different time intervals in XCV and pyridoxine inoculated plants and was found to be significantly induced with different patterns in *B*. *subtilis*-inoculated plants (Fig. 1). After inoculation with *B*. *subtilis*, expression levels of *PDX1*.*2* increased significantly at 48 and 72 hours post inoculation (hpi), in both XCV + *B*. *subtilis* and XCV + *B*. *subtilis* + pyridoxine, inoculated plants. In addition, *B*. *subtilis* and pyridoxine inoculation showed more than 5-fold increase than those in XCV inoculated plants at 72 hpi. Moreover, the transcript abundance of *PDX1*.*3* in *B*. *subtilis* and pyridoxine inoculated plants significantly increased more than 12-fold higher than those in XCV inoculated plants at 72 hpi. *PDX2* transcripts reached approximately 2.3-fold (XCV + *B*. *subtilis*) and 2.6-fold (XCV + *B*. *subtilis* + pyridoxine) increased over the pathogen control at 48 hpi, and then declined, reaching control expression level at 72 hpi. The results affirm that *B*. *subtilis* inoculation is responsible for the overexpression of VitB6 biosynthetic genes. *PDX1*.2 and *PDX1*.3 expression profiling show an escalated expression of pyridoxine at 72 hpi. On the contrary, *PDX2* depict the overexpression in 72 hpi showing differential regulation. *SOS4* gene expression profiling demarcates to nullified results without any significant outcomes. *PDX1*.2, *PDX1*.*3*, *PDX2* genes overexpression confirms the authentic involvement of the *de novo* pathway of VitB6 biosynthesis in tomato upon XCV infection. On the other hand, *SOS4* involving in salvage pathway was not significantly identified for expression patterns questioning how salvage pathway could possibly play a definitive regulatory role in oxidative stress tolerance.

**Figure 2:**
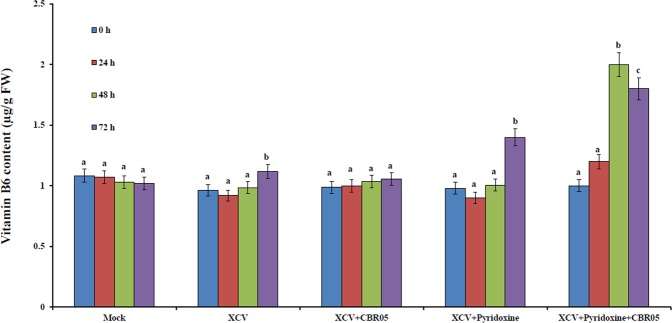
Vitamin B6 contents in plants before and after infection with *Xanthomonas campestris* pv. *vesicatoria*. Data presented in are the means ± SD from three independent experiments and different letters above the columns indicate significant differences at *P* ≤ 0.05 level.

VitB6 contents in the plants without XCV inoculation and with XCV were examined to assess *B*. *subtilis*-induced expression of the VitB6 biosynthetic genes. VitB6 contents in pathogen-inoculated plants were lower than that in the mock-inoculated plants, showing reduction of 14, 6, and 15% for XCV, XCV + *B*. *subtilis*, and XCV + pyridoxine, respectively, at 24 hpi, as compared to those of mock-inoculated plants. These results, in consort with the fact that the transcript abundance of VitB6 gene in tomato after infection by XCV, show that reduced biosynthesis and hence reduced VitB6 content. It might be a natural defense response of plants to pathogenic infection as a part of defense mechanism. However, the VitB6 contents in the XCV + pyridoxine + *B*. *subtilis* inoculated plants, showing increase of 12, 93 and 76% at 24, 48 and 72 hpi, respectively, compared to that of mock-inoculated plants (Fig. 2). The result clearly shows that *Bacillus* inoculation has roles in VitB6 biosynthesis in all intervals indicating a way for profiling the specific genes. Differential regulation of the VitB6 genes could also involve a crosstalk between *de novo* and salvage pathway genes. Hence, further resistance mechanisms could positively associate with antioxidant profiling for establishing prominent disease resistance strategies.

### XCV effect on superoxide dismutase (SOD) and catalase (CAT) activity

**Figure 3:**
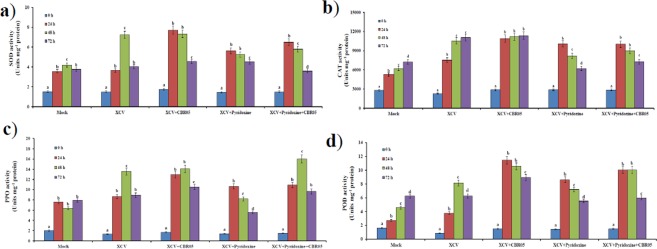
Effect of *Xanthomonas campestris* pv. *vesicatoria* inoculation on antioxidant activity in leaves of tomato. (**a**) SOD activity, (**b**) CAT activity, (**c**) PPO activity, (**d**) POD activity. Means with different letters are significantly different at P ≤ 0.05. Bars represent standard deviations (n = 3).

As shown in Fig. 3a, *B*. *subtilis* + XCV inoculated plants and XCV + pyridoxine inoculated plants had a significant increase in SOD activity at 24 hpi as compared to mock-inoculated plants (115 and 57%, respectively) and XCV inoculated plants (111 and 54%, respectively). We also found significant increase in SOD activity in the XCV + pyridoxine + *B*. *subtilis* inoculated plants, compared them with those in mock-inoculated pants (82%) and XCV inoculated plants (54%), at 24 hpi followed by decrease in SOD activity at 48–72 hpi (Supplementary Table S1). However, the activity of CAT in tomato plants increased rapidly after pathogen inoculation. CAT activity was significantly higher in the XCV + *B*. *subtilis*, XCV + pyridoxine and XCV + pyridoxine + *B*. *subtilis* by 44, 34 and 34%, respectively at 24 hpi, compared to those in XCV inocualted plants (Fig. 3b). We did not find any significant difference in the SOD and CAT activity at 48 hpi both in *B*. *subtilis* and *X*. *campestris* inoculated plants. Reduced levels of SOD and CAT activity was observed in XCV and pyridoxine inoculated plants at 48–72 hpi.

### XCV effect on polyphenol oxidase (PPO) and peroxidase (POD) activity

**Figure 4:**
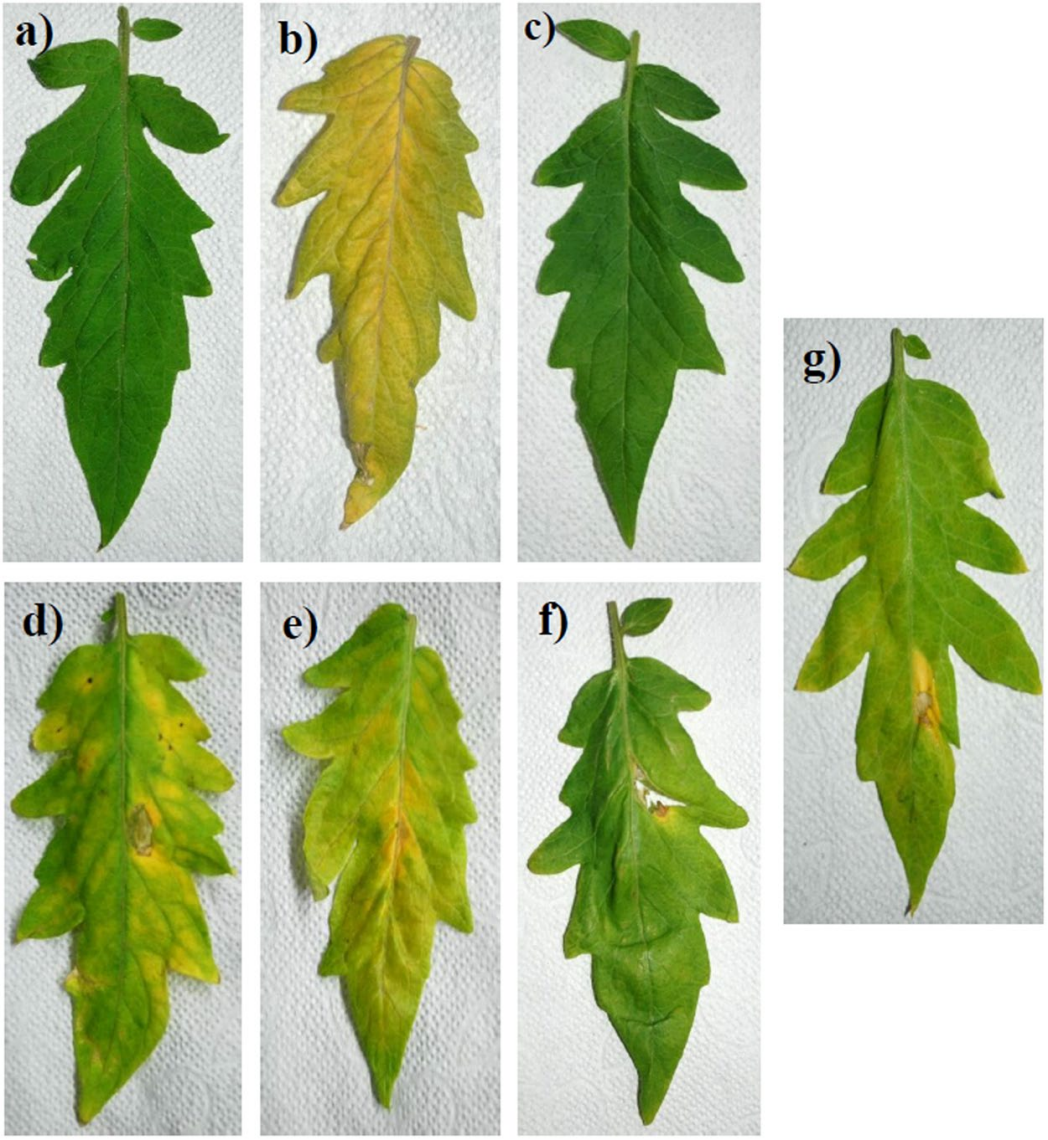
Inoculation results of different tomato plants leaf. (**a**) Leaf of a mock-inoculated plant. (**b**) Leaf of 100 mM Pyridoxine inoculated plant. (**c**) Leaf of *Bacillus subtilis* CBR05 inoculated plant. (**d**) Leaf of *Xanthomonas campestris* pv. *vesicatoria* inoculated plant. (**e**) Leaf of *Xanthomonas campestris* pv. *vesicatoria* and 100 mM pyridoxine inoculated plant. (**f**) Leaf of *Xanthomonas campestris* pv. *vesicatoria* and *Bacillus subtilis* CBR05 inoculated plants. (**g**) Leaf of *Xanthomonas campestris* pv. *vesicatoria*, *Bacillus subtilis* CBR05 and 100 mM pyridoxine inoculated plants.

**Figure 5:**
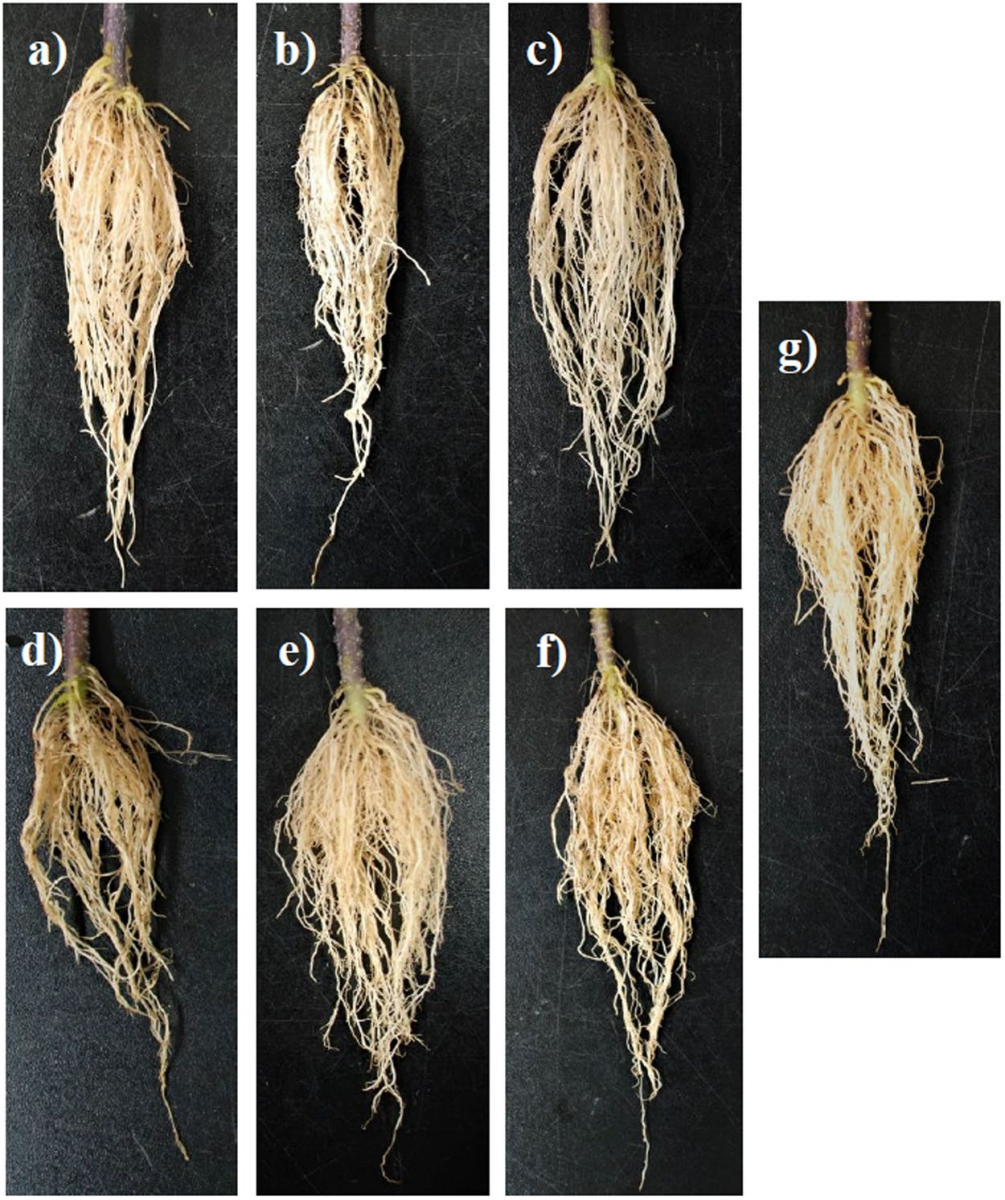
Inoculation results of different tomato plants root. (**a**) The root of a mock-inoculated plant. (**b**) Root of 100 mM Pyridoxine inoculated plant. (**c**) Root of *Bacillus subtilis* CBR05 inoculated plant. (**d**) Root of *Xanthomonas campestris* pv. *vesicatoria* inoculated plant. (**e**) Root of *Xanthomonas campestris* pv. *vesicatoria* and 100 mM pyridoxine inoculated plant. (**f**) Root of *Xanthomonas campestris* pv. *vesicatoria* and *Bacillus subtilis* CBR05 inoculated plants. (**g**) Root of *Xanthomonas campestris* pv. *vesicatoria*, *Bacillus subtilis* CBR05 and 100 mM pyridoxine inoculated plants.

Patterns of increase in PPO activity in the leaves during 24–72 hpi are illustrated in Fig. 3c. In, *B*. *subtilis* + XCV inoculated plants had a noteworthy increase in PPO activity at 48 hpi as compared to mock-inoculated controls (120%), XCV alone (4%), and XCV + pyridoxine (42%) inoculated plants. However, the PPO activity was significantly higher in the XCV + pyridoxine + *B*. *subtilis* inoculated plants, showing an increase of 18% compared with that in the XCV inoculated plants at 48 hpi. As shown in Fig. 3d, the treatment groups had a significant increase in POD production as compared to mock inoculated plants at 24 and 48 hpi. In, *B*. *subtilis* inoculated plants had a significant increase in POD activity at 24–72 hpi as compared to XCV, pyridoxine, and mock-inoculated controls. Induction of POD activity in the leaves was observed during 24 hpi and followed by a decline at 48–72 hpi. At 48 hpi, XCV inoculated plants showing an increase of 78% total POD activity than the mock-inoculated healthy control. Pot trials also corroborate that *B*. *subtilis* compared to pyridoxine supplementation alone show biocontrol activities on the leaf (Fig. 4). Figure 5 shows that root length of *B*. *subtilis* and pyridoxine inoculated plants were greatly increased by co-inoculation. Among inoculation, XCV + pyridoxine + *B*. *subtilis* (23.5 cm), followed by *B*. *subtilis* + XCV (16.5 cm), XCV + pyridoxine (16.0 cm) and mock-inoculated plants (14.0 cm) showed an increase in root length. Whereas, XC inoculated plants showed decreased in root length (9.0 cm).

## Discussion

VitB6 is a collective term for a group of six interconvertible compounds^1,7^. It is most notable for its contribution to amino acid, carbohydrate and fatty acid metabolism where it serves as a cofactor for enzymes^48^. Despite these facts, the effect of VitB6 on plant development, the mechanisms controlling its biosynthesis and stress tolerance are still poorly understood. Reactive oxygen species (ROS) quenching and antioxidant properties by VitB6 have been an extensively studied phenomenon. VitB6 vitamers have been affirmed as significant singlet oxygen quenchers *in vitro*^49^. Nevertheless, well documented in *A*. *thaliana*, it needs to be assessed for its definitive role^50^. Further, PDX1.2 serves its role in sustaining VitB6 under critical conditions and stabilizes PDX1s under abiotic stress^51^. The literature available for most of the crop plants shows the manifestation of both *de novo* and salvage pathways in VitB6 genes for enhanced disease resistance upon phytopathogens infection. Recently, Zhang *et al*.^52^ proved that *Botrytis cinerea-*infected tomato plants show the involvement of the *de novo* vitB6 biosynthetic pathway and not salvage pathway by affirming *SlPDX1*.*2* and *SlPDX1*.*3* genes but not *SlSOS4* through gene silencing studies based on disease severity. Also, *Ralstonia solani RsolPDX1*, *RsolPDX2*, and *RsolPLR* were the three principal factors involved in the VitB6 pathway in *R. solani* AG3^53^. Most of the literature pertaining to VitB6 biosynthesis addresses *de novo* pathway mostly but salvage pathway at rare circumstances, interestingly lack of introns were denoted for plant-pathogen induced response for synthesis^18^. Hence, we could not clearly find a gap in abridging salvage pathway in oxidative stress-induced plant defense and antioxidant properties. The present study further adds novel results for the environmentally useful bacteria in eliciting a plethora of optimal outcomes. A non-native DXP-dependent VitB6 pathway in *B*. *subtilis* for the production of pyridoxine was shown to be present earlier^54^. Till date, the role of *B*. *subtilis* for the production of pyridoxine or involvement in VitB6 biosynthesis have not been classically addressed. It has been made possible through altered mechanisms like metabolic engineering strategies^54^. The present study provides molecular insights into the cumulative plant growth promotion, ISR and *de novo* pathway involvement of VitB6 biosynthesis.

Transcript profiling indicates overexpression of all four VitB6 biosynthetic genes was increased under stress; whereas, differential expression patterns were observed. *PDX1*.2, and *PDX1*.3, were found to be overexpressed significantly after 72 hpi with pyridoxine and *B*. *subtilis* in XCV inoculated plants showing that optimal synthesis of VitB6 could be possibly corroborated to the involvement of *de novo* pathway in tomato after XCV. Most significant upregulation was observed in the transcript profile of *PDX1*.*3*, which showed more than 12-fold increase in expression at 72 hpi. We also found significant up regulation of *PDX2* in XCV + B. *subtilis* and XCV + pyridoxine + *B*. *subtilis* inoculated plants, which were 3.054- and 3.44-fold, respectively, at 48 hpi. From our results, we can clearly infer that *SOS4* expression has not been significant. Increased expression of *PDX1*.*3*, *PDX1*.*3*, and *PDX2* levels in our study proves that *B*. *subtilis* inoculated plants has roles in balancing the VitB6 biosynthesis. The previous study also showed that in *A*. *thaliana*, *PDX2* balancing B6 vitamer levels^55^. This possibly explains that *de novo* pathway predominates over the salvage pathway having mechanisms as a bypass. The absence of a salvage pathway may be due to a demarcated pathway as that of the non-native pathway. In a similar study by Torky^56^, it clearly shows that VitB6 primes defense response and disease resistance in *Capsicum annum* upon Tobacco Mosaic Virus (TMV) infection. We also hypothesize that *B*. *subtilis* plays similar roles in the emancipation of cohesive response as that of TMV. In addition, VitB6 contents in XCV inoculated plants slightly decreased compared to those in the mock-inoculated plants. Moreover, a slight increase in the VitB6 content in mock-inoculated tomato leaves may be responsive to stress or wounding caused by mock infiltration. These results indicate that expression of VitB6 biosynthetic genes in tomato down-regulated after infection with XCV and hence reduced VitB6 content might be a natural response of plants to pathogenic infection as a part of defense mechanism^18^.

Previous studies showed thatVitB6 vitamers can act as antioxidant in plants and may act as an important modulator of redox status during pathogen defense response^12,18,49^. When compared with those in the control plants, increased disease level and increased accumulation of SOD after infection with XCV was observed. The increase of SOD activity accelerated enzymatic conversion of the superoxide anion to H_2_O_2_ and led to further accumulation of H_2_O_2_ in the XCV inoculated plants. Hence, increased accumulation of H_2_O_2_ will lead to production of CAT and POD. We also found that activity of PPO was induced in XCV inoculated plants compared to those in the mock-inoculated control plants. Moreover, antioxidant activity was higher in *B*. *subtilis* inoculated compared to those of other treatments. Previous studies, the showed that enhanced activities of antioxidant enzymes in plant tissues are positively associated with ISR and plant disease suppression^30,37,41^. Moreover, antioxidant activity in mock-inoculated healthy control was consistently present in all the test plants. The enzyme activity in control plants increased over time after inoculation showing native antioxidant enzyme presence.

Further, tomato plants infiltrated with XCV showed increased disease symptoms compared to those infiltrated with biocontrol agent, *B*. *subtilis* CBR05. However, co-inoculation with *B*. *subtilis* showed increased plant growth and decreased disease severity. At the same time supplementation of VitB6 vitamers like pyridoxine show comparatively low levels in the VitB6 synthesis and plant growth properties. Our results showed that the pyridoxine treated plants appeared in yellow color. Thus, the changed coloration may result from changed pigmentation^12^. Moreover, leaves infiltrated with pyridoxine and XCV increased the severity of chlorosis and necrosis associated with disease. Further inoculation of pyridoxine with *B*. *subtilis* colonizes root in plants and expresses root parameters. These results suggest that a possible role of pyridoxine in promoting root cell division and elongation. In our study, it clearly shows the above phenomena wherein, *B*. *subtilis* CBR05 extensively promote root growth in length and altered health in tomato roots through induced systemic resistance^57^. Further, active principles in *B*. *subtilis* have been attributed to surfactin in wheat as an elicitor^58^. With this view, active principles of *B*. *subtilis* could possibly act as a launch pad for the development of novel elicitors from our pilot study. Interestingly it has been observed that *B*. *subtilis* remarkably show similar patterns of 100 mM pyridoxine supplementation. Our proposition stating *B*. *subtilis* to be adaptable tool invoke several questions for the application in field conditions. Hence, pot culture experiments guarantee the application modalities in plants. The omnipresence of *B*. *subtilis* owing to several competencies in suppressing plant pathogens has been extensively studied^39–41^. *B*. *subtilis* has been well documented for usage against *Fusarium* wilt, *Sclerotina* *sclerotiorum*, *Pythium*, *Phytophthora*, *Rhizoctonia*, *Septoria*, and *Verticillium* in tomato^59^. This was further characterized at the molecular level showing a distinct lineage for the *B*. *subtilis* with copious prominent roles in plant enhancement mechanisms. XCV infection in Chinese cabbage has been studied for plant growth promotion and ISR which indicate *Bacillus* sp. of predominance^60^. Nevertheless, *Xanthomonas* abatement along with numerous molecular dissection mechanisms and VitB6 biosynthesis is lacking. Our study makes this versatile *B*. *subtilis* to be utilized as a broad spectrum agriculturally important microorganism. The present study is a holistic approach to address the *B*. *subtilis* eluding XCV with multiple molecular insights.

Current research focuses on assorted approach in interpreting real-time expression patterns of *B*. *subtilis* CBR05 induced VitB6 biosynthetic genes against XCV in tomato. The advancement in identifying *B*. *subtilis* CBR05 is the inherent objective of the present study. *PDX1*.*2*, *PDX1*.*3*, and *PDX2* are the overexpressed VitB6 biosynthetic genes denoting involvement of *de novo* pathway in VitB6 biosynthesis induced by *B*. *subtilis* CBR05 against XCV. However, genes involved in salvage pathway have been not regulated as like *de novo* pathway genes. This is the first report that *B*. *subtilis* induces expression of VitB6 biosynthesis and can be a versatile bacterium in multifarious roles in tomato plant upgraded productivity. The present study can be rationalized for any crop and VitB6 biosynthesis.

## Materials and Methods

### Microorganism and culture conditions

In the present study, *Xanthomonas campestris* pv. *vesicatoria* (XCV) KACC11154 obtained from Korean Agricultural Culture Collection (KACC), South Korea, was used as the phytopathogen. *B*. *subtilis* CBR05 used in this study were obtained from Prof. Se Chul Chun lab, used as a biocontrol agent. Bacterial cells were subcultured in tryptic soy broth. They were transferred to tryptic soy agar and incubated at 30 °C for 24 h. The bacterial cells were harvested and resuspended in 10 mM MgCl_2._ *B*. *subtilis* CBR05 viable population was adjusted to 10^8^ CFU/ml for further studies.

### Plant materials and growth conditions

We obtained seeds of tomato from Korea seed resource center. They were surface sterilized with 70% ethanol for 5 min, finally rinsed three times with sterile distilled water. Tomato seedlings were transferred into plastic pots after four weeks. The experiment was carried out in a randomized complete block design with three plants as replicates in each of the following treatments: (i) Mock (4% maltose and 1% peptone solution) (ii) XCV alone (iii) XCV + *B*. *subtilis* (iv) XCV + 100 mM pyridoxine (v) XCV + *B*. *subtilis* + 100 mM pyridoxine. Pots were incubated in a growth room. Three pots were maintained per treatment, each with a single plant and arranged in a completely randomized design with three replications.

### Total RNA isolation and cDNA synthesis

Leaf samples were collected from tomato plants. Total RNA was isolated from frozen tomato tissue using the RNeasy Plant Mini Kit (Qiagen, Germany), according to manufacturer’s instructions. Genomic DNA contamination from RNA samples was removed by DNAse treatment. Formaldehyde agarose gel electrophoresis was used for the verification of the integrity of total RNA. We determined the purity and concentration of the total RNA by spectrophotometry (NanoDrop ND-1000 Spectrophotometer; Celbio, Italy). RNA samples were reverse transcribed into cDNA using First-strand cDNA synthesis kit (Bioneer, Korea).

### Quantitative PCR (qPCR)

The synthesized cDNA was used as a template for real-time PCR reactions using the instrument CFX96™ Real-Time System (Bio-Rad, Hercules, CA, USA). The sequences of primers used in this study for RT-qPCR analysis were listed in Supplementary Table S2. Each reaction (25 μL) contained 12.5 μL of AccuPower® 2x Greenstar qPCR Master Mix (Bioneer, Korea), forward and reverse primer (0.4 μM), 5 μL of diluted cDNA sample and nuclease-free water. The qPCR reactions were carried out using the following PCR cycling conditions: 95 °C for 10 min (1 cycle), the 20 s at 95 °C followed by annealing and extension at 60 °C for 45 s (40 cycles). The actin gene was used as the internal reference for normalization. The expression level of the VitB6 gene transcripts was calculated using CFX Manager Software (Bio-Rad). All samples were analyzed with three independent total RNA samples.

### Measurement of VitB6 content

VitB6 contents were determined using a bioassay with Yeast strain (*Saccharomyces cerevisiae* ATCC9080), auxotrophic for VitB6. Leaf samples were collected from each experiment and leaf extracts were prepared with a protocol as described earlier^18^. Cells were grown in Pyridoxine Y medium for 12 h at 30 °C, 220 rpm. Overnight culture was washed two times with Pyridoxine Y medium for removal of residual pyridoxine. One milliliter of leaf extract was added to tubes containing 5 × 10^8^ yeast cells in 5 ml of Pyridoxine Y medium. Assay tubes were incubated for 16 h, at 30 C, 220 rpm. Yeast growth was measured by spectrophotometry at 540 nm. Pyridoxine was used as a standard.

### Determination of antioxidant enzymes

#### Determination of SOD activity

SOD was determined using the SOD activity kit (Enzo Life Sciences Inc., USA), according to manufacturer’s instructions. The enzyme extract (25 μl) was added to 150 μl of Master Mix. The reaction was started by adding 25 μl of 1× xanthine solution to all the wells. The well without enzyme solution (added 25 μl of the 1× SOD buffer) were considered as a control. The total SOD activity was measured using a microtiter plate reader, optical density (OD) at 450 nm. The specific activity was expressed as units per milligram of protein (Units mg^−1^ protein).

#### Determination of CAT activity

The catalase fluorometric detection kit (Enzo-Catalog # ADI-907-027) used to detect CAT activity by measuring the amount of H_2_O_2,_ according to the manufacturer’s protocol. The reaction mixture contained 50 μL of enzyme extract and 50 μL of 40 μM H_2_O_2_. They were incubated at room temperature for 60 min. After incubation, 100 μL of reaction cocktail was added to the mixture and incubated plate for 15 min. The consumption of H_2_O_2_ was monitored using excitation at 570 nm and measured fluorescence. The specific activity was expressed as Units mg^−1^ protein.

#### Determination of PPO activity

PPO activity was determined spectrophotometrically. The reaction mixture consisted of 200 μL enzyme extract and 1.5 mL of 0.1 M sodium phosphate buffer (pH 6.5). We added 200 μL of 0.01 M catechol to the reaction mixture. The enzyme activity was measured at 420 nm. The specific activity was expressed as Units mg^−1^ protein.

#### Determination of POD activity

POD activity was quantified by mixing the enzyme extract (50 μL) with 2.85 mL of 100 mM phosphate buffer (pH 7.0) and 20 mM guaiacol (50 μL). Followed by addition of 40 mM H_2_O_2_ (20 μL) to the reaction mixture. The oxidation reaction was measured by spectrophotometry at 470 nm. The specific activity was expressed as Units mg^−1^ protein.

### Statistical analysis

All the experiments were conducted in triplicate and results were tabulated as the mean ± standard deviation (SD). Data were analyzed by analysis of variance (ANOVA). Student’s t-test, Duncan’s Multiple Range Test, and the probability values of *P* ≤ 0.05 were considered to be significant.

## Supplementary information

Supplementary information
