# Supplementary material for: Bacillus subtilis CBR05 induces Vitamin B6 biosynthesis in tomato through the de novo pathway in contributing disease resistance against Xanthomonas campestris pv. vesicatoria
Source: Sci Rep. 2019 Apr 24;9:6495. doi: 10.1038/s41598-019-41888-6 (PMC6482200; doi:10.1038/s41598-019-41888-6)
Supplement: Supplementary file 1 — Supplementary information [file 41598_2019_41888_MOESM1_ESM.pdf]

Bacillus subtilis* CBR05 induces Vitamin B6 biosynthesis in tomato through the *de novo* pathway in contributing disease resistance against *Xanthomonas campestris* pv. *vesicatoria

Running Head: *B. subtilis* induced expression of VitB6 biosynthetic genes

Murugesan Chandrasekaran[1], Manivannan Paramasivan[2], and Se-Chul Chun [3]*

[1]Department of Food Science and Biotechnology, Sejong University, 209 Neungdong-ro, Gwangjin-gu, Seoul 05006, Republic of Korea. Email address: chandrubdubio@gmail.com

[2]Department of Microbiology, Bharathidasan University, Tiruchirappalli 620024, Tamilnadu, India. Email address: manivannan3065@gmail.com

[3]Department of Environmental Health Science, Konkuk University, 120 Neungdong-ro, Gwangjin-gu, Seoul 05029, Republic of Korea. Email address: scchun@konkuk.ac.kr

**Corresponding author*

Se-Chul Chun,

Department of Environmental Health Science,

Konkuk University, Seoul, Republic of Korea.

Phone: +82-2-450-3727

Fax: +82-2-450-3726

E-mail: scchun@konkuk.ac.kr

Table S1. Antioxidant enzyme activities

Treatment	Enzyme activities				
	**Time**	**SOD (Units mg^{-1} protein)**	**CAT (Units mg^{-1} protein)**	**PPO (Units mg^{-1} protein)**	**POD (Units mg^{-1} protein)**
Mock	0 h	1.51 ± 0.03^{g}	2802.26 ± 0.54^{i}	1.98 ± 0.21^{g}	1.65 ± 0.01^{j}
	24 h	3.58 ± 0.02^{f}	5261.97 ± 0.65^{h}	7.60 ± 0.02^{e}	2.71 ± 0.03^{i}
	48 h	4.20 ± 0.06^{e}	6224.27 ± 1.30^{g}	6.38 ± 0.09^{f}	4.58 ± 0.01^{g}
	72 h	3.75 ± 0.02^{f}	7217.17 ± 1.24^{f}	7.91 ± 0.05^{e}	6.24 ± 0.01^{f}
XCV	0 h	1.50 ± 0.03^{g}	2274.16 ± 0.83^{j}	1.32 ± 0.01^{gh}	0.86 ± 0.01^{jk}
	24 h	3.65 ± 0.06^{f}	7524.70 ± 0.59^{f}	8.65 ± 0.05^{d}	3.78 ± 0.05^{h}
	48 h	7.25 ± 0.04^{ab}	10540.97 ± 0.93^{de}	13.56 ± 0.03^{b}	8.14 ± 0.03^{d}
	72 h	4.02 ± 0.01^{ef}	11067.58 ± 0.70^{c}	8.94 ± 0.01^{d}	6.26 ± 0.01^{f}
XCV+CBR05	0 h	1.75 ± 0.01^{g}	2861.01 ± 0.24^{i}	1.71 ± 0.01^{g}	1.51 ± 0.01^{jk}
	24 h	7.71 ± 0.04^{a}	10893.04 ± 0.93^{d}	12.94 ± 0.04^{bc}	11.44 ± 0.04^{a}
	48 h	7.35 ± 0.01^{ab}	11179.15 ± 0.29^{b}	14.07 ± 0.05^{b}	10.55 ± 0.01^{b}
	72 h	4.55 ± 0.01^{e}	11347.46 ± 0.57^{a}	10.53 ± 0.02^{c}	8.93 ± 0.02^{c}
XCV+Pyridoxine	0 h	1.45 ± 0.02^{g}	2870.85 ± 0.39^{i}	1.41 ± 0.05^{gh}	1.43 ± 0.05^{jk}
	24 h	5.62 ± 0.03^{d}	10091.23 ± 0.37^{d}	10.62 ± 0.08^{c}	8.62 ± 0.02^{cd}
	48 h	5.27 ± 0.03^{d}	8218.03 ± 0.16f	8.21 ± 0.06^{de}	7.21 ± 0.01^{e}
	72 h	4.52 ± 0.02^{e}	6225.07 ± 0.41^{g}	5.54 ± 0.06^{f}	5.52 ± 0.04^{fg}
XCV+Pyridoxine+CBR05	0 h	1.52 ± 0.02^{g}	2850.21 ± 0.62^{i}	1.51 ± 0.02^{g}	1.52 ± 0.01^{jk}
	24 h	6.53 ± 0.03^{c}	1008.32 ± 0.43^{de}	10.93 ± 0.03^{c}	10.32 ± 0.00^{b}
	48 h	5.80 ± 0.01^{d}	9001.20 ± 0.79^{e}	16.32 ± 0.00^{a}	10.25 ± 0.01^{b}
	72 h	3.61 ± 0.04^{f}	7254.02 ± 0.32^{f}	9.63 ± 0.02^{d}	6.12 ± 0.02^{f}

Mean ± SD of three replicates within a column followed by the different letters indicate a significant difference at $P \leq 0.05$.

Table S2. List of primers used in the study

**Gene name**	**Host plants**	**Primer name**	**Primer sequence (5’ 3’) →**
Actin	*S. lycopersicum*	*Actin-F*	AGG CAC ACA GGT GTT ATG GT
		Actin-R	AGC AAC TCG AAG CTC ATT GT
PDX1.2	*S. lycopersicum*	*PDX1.2-F*	GAT GCA GCT GGG TTG TGA TG
		PDX1.2-R	TCC AAA CCA CTG CTA GCC GC
PDX1.3	*S. lycopersicum*	*PDX1.3-F*	CAT GTG CGT TCC GTT ATG GG
		PDX1.3-F	TGA ACC ACA GGG AGC CTA CC
PDX2	*S. lycopersicum*	*PDX2-F*	CAA AGC TTC GGA ACG CGT TCA A
		PDX2-R	GTC AAT GAG TAG CCA TTT GAC C
SOS4	*S. lycopersicum*	*SOS4-F*	GCG TAT TTC ACG GGA ACT GG
		SOS4-R	CTT GAG ACA GCA AGC TCT GC
